# Network Topology Reveals Key Cardiovascular Disease Genes

**DOI:** 10.1371/journal.pone.0071537

**Published:** 2013-08-15

**Authors:** Anida Sarajlić, Vuk Janjić, Neda Stojković, Djordje Radak, Nataša Pržulj

**Affiliations:** 1 Department of Computing, Imperial College London, London, United Kingdom; 2 Institute for Cardiovascular Disease “Dedinje,” University of Belgrade, Belgrade, Serbia; King’s College, London, United Kingdom

## Abstract

The structure of protein-protein interaction (PPI) networks has already been successfully used as a source of new biological information. Even though cardiovascular diseases (CVDs) are a major global cause of death, many CVD genes still await discovery. We explore ways to utilize the structure of the human PPI network to find important genes for CVDs that should be targeted by drugs. The hope is to use the properties of such important genes to predict new ones, which would in turn improve a choice of therapy. We propose a methodology that examines the PPI network wiring around genes involved in CVDs. We use the methodology to identify a subset of CVD-related genes that are statistically significantly enriched in drug targets and “driver genes.” We seek such genes, since driver genes have been proposed to drive onset and progression of a disease. Our identified subset of CVD genes has a large overlap with the Core Diseasome, which has been postulated to be the key to disease formation and hence should be the primary object of therapeutic intervention. This indicates that our methodology identifies “key” genes responsible for CVDs. Thus, we use it to predict new CVD genes and we validate over 70% of our predictions in the literature. Finally, we show that our predicted genes are functionally similar to currently known CVD drug targets, which confirms a potential utility of our methodology towards improving therapy for CVDs.

## Introduction

Understanding the role and function of proteins in diseases is a foremost challenge. Since proteins bind to each other to perform a function, utilizing networks of protein-protein interactions (PPIs) to address this challenge has gained attention. A *network* (also called a *graph*) is a common model of a set of objects (e.g., proteins) and their interactions and hence, graph theoretic approaches, are commonly used for analyzing network data. In a PPI network, nodes correspond to proteins and links between them to physical interactions between the proteins. Topological properties of PPI networks have been studied to extract new disease-related knowledge [1]–[4]. We build on those approaches and focus on cardiovascular diseases to examine a predictive power of similarity in PPI network wiring around proteins involved in these diseases.

Cardiovascular diseases (CVDs) is a group of diseases of the heart and blood vessels and a major global cause of death, with more people dying every year from CVDs than from any other cause [5]. For example, 1 in 3 deaths in the United States is caused by CVDs. Hence, controlling and preventing CVDs and their complex pathogenesis, that is influenced by genetic, environmental and lifestyle factors, have gained considerable attention [5]. CVDs are studied in a mechanistic, genetic and biochemical contexts that include genomic [6], gene expression [7] and proteomic studies [8]. In cardiovascular research, proteomics is used in two ways: for investigating protein function in different physiological and disease processes (mechanistic studies) and for investigating difference in protein levels and function in a diseased state of an organism (biomarker studies) [8], [9]. Proteomics research includes sample pre-processing or sample pre-fractionation, mass spectrometry and data analysis [10].

Integrated research of gene expression and protein-protein interaction (PPI) networks can provide unique benefits to studying molecular machinery of various diseases, including CVDs. There are several studies which employ PPI networks in search for biomarkers of CVDs [11]–[13]. For instance, Camargo and Azuaje [11] constructed a PPI network consisting of human heart failure relevant interactions, which they used to analyse a relationship between gene co-expression and PPI network connectivity. They used Gene Ontology (GO) [14] to establish a relationship between the connectivity of proteins in the PPI network and their involvement in specific disease-related processes. In a later work, they suggested a set of potentially novel Dilated cardiomyopathy signature genes by integrating functional PPI network information and data sets describing gene expression profiles [12]. Jin et al. [13] formed a cardiovascular-related PPI network based on PPI and signal transduction data. They used statistical methods to successfully discover biomarkers in the newly formed network. Zhang et al. [15] introduced a computational method based on six network topological features, and constructed a combined classifier to predict candidate genes for coronary artery diseases.

It has been shown that directly linked proteins in the human PPI network are more likely to cause similar diseases [2], [3]. Also, Goh et al. [16] created a bipartite “diseasome” network, where one partition consists of a set of diseases and the other of a set of disease genes (and where by definition of a bipartite network, all edges in the network go between the partitions). They used it to generate two network projections: disease gene network and human disease network (which they found is clustered according to major disorder classes). By exploring centrality and peripherality of genes in the network, they showed that contrary to essential human genes which encode hub proteins, majority of disease genes do not encode hubs, and are localized in the periphery of the network [16]. Yidirim et al. [17] analyzed a bipartite network composed of drugs and proteins targeted by drugs, linked by drug-target binary associations, with a goal of understanding the properties of drug targets in the context of cellular and disease networks. They used the measure of shortest distance between nodes in the network to find significant differences between etiological and paliative drugs. Radivojac et al. [18] used machine learning to detect gene-disease associations. They based their approach on the PPI network, protein-disease associations, protein sequence, functional annotation, and measure of distance in the protein interaction network. Goldenberg et al. [19] used gene and gene-product interaction network trying to identify genes that play important role in initiation and progression of lung cancer. They identified a small set of influential genes, looking into genes whose neighbors show high expression change (in cancerous tissue versus normal) regardless of their own expression.

Several methods have shown that PPI network topology around proteins is a predictor of their function [4], [20], [21]. The method proposed in [20] summarizes the local topology around a protein in a PPI network into a “signature” of a protein, which is a vector containing counts of small subgraphs (“graphlets”) that the protein touches. Then, proteins in the PPI network are grouped based on similarity of their “signatures,” and it has been shown that proteins within those groups belong to same protein complexes, perform the same biological function and are part of the same subcellular components [20]. Also, the same similarity of the wiring (i.e. topology) in the extended neighborhood around a protein in the PPI network was used to predict the involvement of a protein in disease [4], [21]: a series of clustering methods was applied to the proteins with similar PPI network wiring and the obtained clusters were significantly enriched in cancer and disease related proteins. This lead to predictions of new melanogenesis related genes purely from the topology of the human PPI network and the predictions were phenotypically validated [4], [21].

Janjić and Pržulj [22] demonstrate the existence of topologically and functionally homogeneous “core subnetwork” of the human PPI network, which is enriched in disease genes, drug targets, and a small number of genes that have theoretically been proposed to be absolutely required for tumor formation and that are usually referred to as “driver genes” [23]. They call this subnetwork the “Core Diseasome” [22]. They postulate that the Core Diseasome subnetwork is the key to disease onset and progression and hence should be the primary object of therapeutic intervention. They find this subnetwork purely computationally by utilizing the 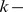
core decomposition algorithm [24], [25] applied to the human PPI network. GRAAL family of network alignment algorithms [26]–[30] uses the wiring around nodes to align topologically similar nodes across different PPI networks. They were utilized to prove that the Core Diseasome, obtained purely by k-core decomposition of the human PPI network, has a unique topology in PPI network.

Hence, it seems that the evolution has constrained the interactome topology so that similar topology is selected for similar biological function. A complete explanation of why is this true is beyond the scope of this study and is a subject of future research. Here, we explore this issue further by examining if it holds for genes implicated in CVDs. This may also lead to improvements in a choice of therapy, which is important given the fact that CVDs are currently a major global cause of death [5].

### This Study

We explore the relationship between the wiring around proteins (we use terms protein and gene interchangeably) in the human PPI network and their involvement in CVDs. In particular, we find clusters of proteins with similar wiring to the proteins already known to be involved in CVDs (see sections Similarity Measure and Clustering Methods). We identify a consensus set of CVD genes from clusters that are statistically significantly enriched with CVD-related genes (see section Similarity Measure). Then, to validate potential gene candidates that might drive CVD onset and progression and are drug targets, we utilize the method of [22] mentioned above (see section The Core of Cardiovascular Diseasome) and find that this consensus set of genes is enriched in drug targets and driver genes (see section The Key Cardiovascular Disease Genes). Furthermore, this consensus set has a large overlap with the Core Diseasome. We also find that many of these genes are functionally similar to known CVD drug targets. Hence, we call this consensus set the *Key CVD Genes* and we use the same methodology to predict new CVD gene candidates. We validate that the predicted genes are functionally similar to currently known CVD drug targets, indicating that our methodology may be used for finding new genes relevant for CVD therapy (see section Therapeutic Properties of Key and Predicted CVD Genes).

This combination of methods has not been used before. Also, no similar methodology has previously been applied to CVD-related genes. It produces highly confident CVD gene predictions, as evident by literature validations and therapeutically relevant functional enrichments (discussed in detail in the Results and Discussion section).

## Methods

**Figure 1:**
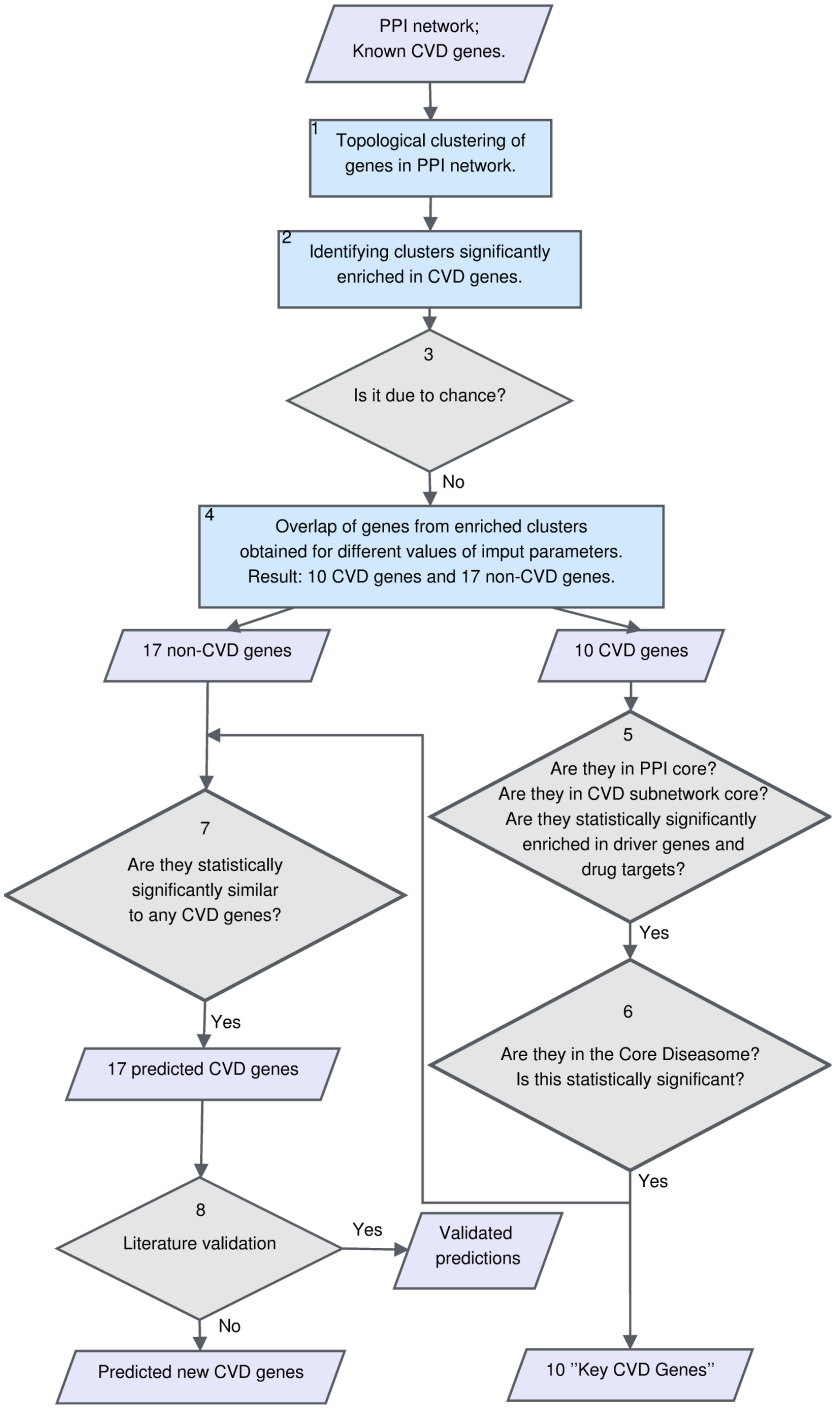
Flowchart of our approach. Parallelograms denote inputs and outputs. Rectangles denote analyses. Rhombuses denote choices to be made.

In this paper we introduce a methodology to identify important CVD genes that could be used to predict new therapeutically relevant CVD genes (shown on the flowchart in Fig. 1). Here, we describe all the steps in more detail.

### Data Sets

We use the latest human PPI network data from I2D, version 2.0.0 (http://ophid.utoronto.ca/), because I2D integrates most of the available PPI data (http://ophid.utoronto.ca/ophidv2.204/statistics.jsp). We remove all self-interactions, as well as any low confidence (originating from only one source) and predicted interactions. To further reduce noise in the PPI network, we remove all proteins with degree lower than 4 (where *degree* is the number of interactors with the protein of interest), since their low connectivity may be a result of a lack of experiments performed for detecting their interactions, i.e. they may be involved in false negative interactions. The resulting human PPI network has 82,649 interactions between 7,551 proteins.

We obtain the list of genes involved in CVDs from two sources to increase coverage: (i) Disease Ontology (DO) Lite (http://django.nubic.northwestern.edu/fundo/) [31] and (ii) pathways from KEGG database (http://www.genome.jp/kegg/), downloaded in September 2012. The list includes genes known to be involved in the following CVDs in DO: aortic-aneurysm, atherosclerosis, brain-ischemia, cardiovascular-disease, cerebrovascular disorder, heart-disease, heart-failure, intermediate-coronary-syndrome, ischemia, moyamoya-disease, pseudoxanthoma-elasticum (which later may result in the form of premature atherosclerosis), stroke, Takayasu’s-arteritis, thrombophilia, thrombophlebitis, vascular-dementia, vascular-disease, and vasculitis. We obtain additional genes from the following KEGG pathways: hypertrophic cardiomyopathy, arythmogenicright ventricular cardiomyopathy, dilated cardiomyopathy, and viral myocarditis. This results in the set of 656 CVD-related genes, out of which we analyze 423 genes that are present in human PPI network.

We download the drug target data from Drugbank (http://http://www.drugbank.ca/): there are 1,245 drug targets in our PPI network, among which 199 are known CVD genes.

### Similarity Measure

**Figure 2:**
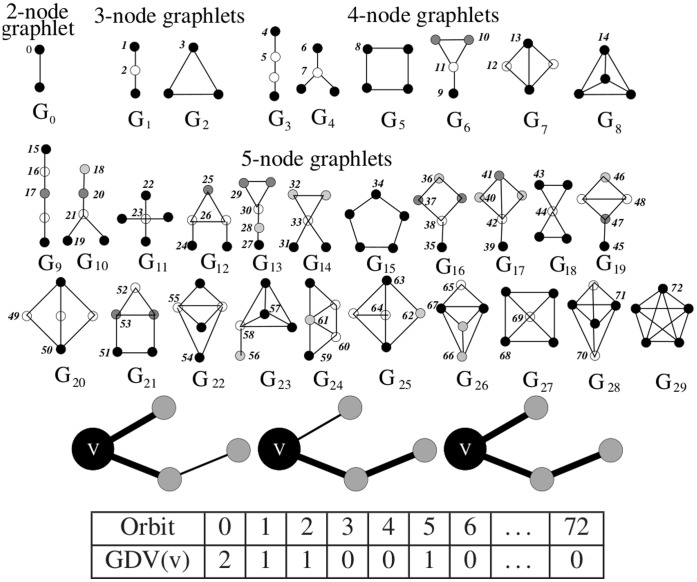
73 Graphlets and Graphlet Degree Vector (GDV) of a node. **Above:** Graphlets with up to five nodes, denoted by 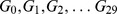. They contain 73 “symmetry groups,” denoted by 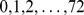. Within a graphlet, nodes belonging to the same symmetry group are of the same shade [33]. **Below:** An illustration of the GDV of node 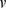. 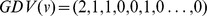, meaning that 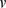 is touched by two edges (orbit 0), illustrated in the left panel, an end-node of one graphlet 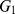 (orbit 1), illustrated in the middle panel, the middle node of one graphlet 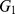 (orbit 2), illustrated in the left panel again, no nodes of a triangle (orbit 3 in graphlet 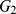), no end-node of graphlet 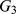 (orbit 4), one middle node of graphlet 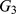 (orbit 5), illustrated in the right panel, and no other orbits [22]-Reproduced by permission of The Royal Society of Chemistry (http://pubs.rsc.org/en/content/articlehtml/2012/mb/c2mb25230a).

As stated above, a *network* (also called a *graph*) is a set of nodes that are linked by edges. *Graphlets* are small connected non-isomorphic induced subgraphs of a network [32] (denoted by 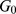 to 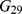 at the top of Fig. 2). To find proteins in a network with similar wiring around them, we use the similarity measure introduced in [20]. This similarity measure is a generalization of the degree of a node and it counts the number of all two to five node graphlets that a node touches, taking into account different “symmetry groups” within each graphlet (numbered from 0 to 72 at the top of Fig. 2, introduced in [22]). For example, it is topologically relevant whether a node touches graphlet 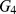 at the middle node, or at one of the end nodes (top of Fig. 2). These counts are coordinates in the 73-dimensional *Graphlet Degree Vector (GDV)* of a node (detailed in [33]). An illustration of a GDV of node 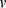 is given at the bottom of Fig. 2, introduced in [22].

We compute the similarity between GDVs of nodes 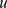 and 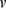 in graph 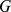 as follows [20]. If 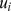 is the 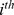 coordinate in the GDV of node 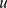, and 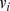 is the 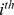 coordinate in the GDV of node 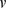, than the distance between these two coordinates is computed as:
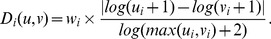
(1)


In formula (1), 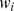 represents the weight of coordinate 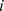, which takes into account dependencies between orbits, as described in [20]. The total distance between GDVs of nodes 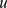 and 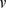, normalized in 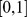 range, is calculated as:
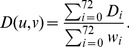
(2)


Finally, *GDV similarity* of the two nodes is computed as:
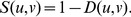
(3)


As mentioned above, GDV similarity between proteins in the human PPI network has already been used to successfully predict protein function and involvement in disease [4], [20], [21], [34]. Here, we examine its usability for predicting CVD-related genes. We use it to make clusters of proteins with similar wiring in the PPI network (see below).

### Clustering Methods

By using the above described GDV similarity between proteins in the human PPI network, we obtain clusters of proteins with similar wiring around them in the PPI network. Clustering is a hard problem and a major research area in its own. Some clustering methods, such as K-nearest neighbours(KNN), produce overlapping clusters, while others, such as K-medoids, or Hierarchical clustering, produce clusters with non-overlapping sets of elements. We use a method that produces non-overlapping clusters to avoid enrichments in clusters that are due to cluster overlap. Since the choice of the best clustering method is heavily data dependent, we try two methods described below (step 1. in Fig. 1).

Note that a success of a particular clustering method depends on the data and can be different for different networks [35]. Discussing the reasons for different performance of different clustering methods is beyond the scope of this paper.

#### Hierarchical Clustering (HIE)

This method creates a dendogram that represents a cluster tree, which is a multilevel hierarchy meaning that clusters at one level of the hierarchy are joined into a cluster at the next level. The process of creating clusters starts by assigning each node to its own cluster and follows by finding the “closest” pair of clusters to merge into a single cluster. Recall that, we specify the closeness between a pair of nodes by their GDV similarity. If there are many closest pairs, a single pair is chosen randomly. Then, we compute the “closeness” between the newly formed cluster and each of the old clusters as the average of GDV similarities between the nodes of the clusters. Again, the closest pair of clusters is merged into a single cluster. This process repeats until all nodes are clustered into one cluster. In order to create the desired number of disjoint clusters it is necessary to cut the hierarchical tree at some point. We denote the minimal number of clusters that are obtained with a cut by 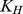.

#### K-medoids Clustering (KM)

A *medoid* is a node in a cluster whose average distance to all other nodes in the cluster is minimal. The algorithm randomly picks 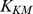 nodes as cluster medoids and assigns all remaining nodes to 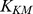 clusters. Each node is assigned to the cluster with the medoid minimally distant from the node in question. Ties are broken randomly. Then, in each cluster, a new medoid node is found with respect to the nodes of the cluster. All non-medoid nodes in the network are then reassigned to new 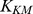 clusters with these new medoids. These steps are repeated until the same set of nodes is chosen as cluster medoids.

### Statistical Significance

For each cluster obtained by using each of the clustering methods described above, we compute the enrichment in CVD-related proteins (or equivalently, genes). We compute statistical significance (*p*-value) of obtaining this or higher enrichment purely by chance. The *p*-value is computed in a standard way, by using the hypergeometric cumulative distribution as follows. We denote the number of genes in the human PPI network with 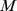, the number of genes that are involved in CVDs with 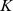, and the size of the cluster in question with 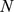. The *p*-value, or the probability that 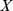 or more disease genes will be found in the cluster by chance, is computed as follows:
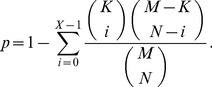
(4)


We apply *Benjamini-Hochberg false discovery rate* (FDR) correction [36] on the resulting *p*-values in order to take into account a possibility of obtaining significant *p*-values in a large number of experiments purely by chance. We report such corrected *p*-values. Sensible cut-offs for *p*-values are in the range from 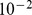 to 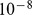 [37]. We use the *p*-value of 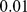 as a cut-off to define clusters statistically significantly enriched in CVD-related genes.

First, we apply Hierarchical clustering to our PPI network. In different runs of the algorithm, we choose the minimum number of resulting clusters 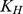 to be: 50, 75, 100, 200, 500, 700, 1000 and 2000. These numbers are chosen to cover different sizes of clusters in order to identify the optimal size at which the enrichment in CVD genes would occur. Unfortunately, the obtained clusters were not statistically significantly enriched with CVD genes, indicating that HIE can not be used for obtaining clusters of CVD-enriched genes purely from the topology of the PPI network.

KM method produced clusters of proteins statistically significantly enriched in CVD genes. The number of medoids, and therefore clusters, 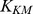, that we use are: 50, 75, 100, 200, 300, 500, 700 and 1000. 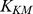 larger than 1000 caused clusters to be too small for any statistical analyses. The obtained clusters depend on the initial random choice of medoids, as previously explained. Hence, for each value of 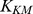 mentioned above, we repeat the experiment five times. To increase coverage, we take a union of genes that are found in statistically significantly enriched clusters for all five experiments per choice of 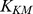
(step 2. in Fig. 1). As a result, in CVD enriched clusters we identify following gene sets:

- For 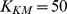: 86 CVD genes and 572 non-CVD genes;
- For 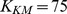: 48 CVD genes and 282 non-CVD genes;
- For 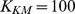: 54 CVD genes and 282 non-CVD genes;
- For 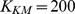: 75 CVD genes and 277 non-CVD genes;
- For 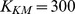: 13 CVD genes and 40 non-CVD genes;
- For 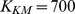: 17 CVD genes and 23 non-CVD genes.

To find the “most important” CVD genes, we apply an additional filter: we seek CVD genes that are in the intersection of the above gene sets, obtained from statistically significantly enriched clusters for different values of 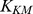
(step 4. in Fig. 1). We find 10 such genes (listed in Table 1)and analyse them further (see below).

**Table 1:** The Ten Key Cardiovascular Disease Genes.

Entrez ID	Gene name	GO term	Cardiovascular disease (CVD)
25	ABL1	Intracellular signaling cascade (BP),Signal transducer activity (MF)	Viral myocarditis.
6464	SHC1	Intracellular signaling cascade (BP),Signal transducer activity (MF)	Atherosclerosis.
6667	SP1	Enzyme binding (MF)	Trombophlebitis.
367	AR	Intracellular signaling cascade (BP),Intracellular receptor-mediatedsignaling pathway (BP), Signaltransducer activity (MF)	Atherosclerosis.
1499	CTNNB1	Intracellular signaling cascade (BP),Intracellular receptor-mediatedsignaling pathway (BP), Enzyme binding (MF),Signal transducer activity (MF)	Arythmogenic right ventricular cardiomyopathy (ARVC).
2534	FYN	Intracellular signaling cascade (BP)	Viral myocarditis.
60	ACTB	Enzyme binding (MF)	Arythmogenic right ventricular cardiomyopathy(ARVC), Hypertrophic cardiomyopathy (HCM), Viral myocarditis, Dilated Cardiomyopathy (DCM).
10014	HDAC5		Heart failure.
1956	EGFR	Intracellular signaling cascade (BP),Enzyme binding (MF), Signal transduceractivity (MF)	Trombophlebitis, Stroke.
2099	ESR1	Intracellular signaling cascade (BP),Intracellular receptor-mediatedsignaling pathway (BP), Signaltransducer activity (MF)	Stroke, Atherosclerosis, Cerebrovascular disorder.

The first two columns: ten Key CVD genes (Entrez Gene IDs and Official Gene Symbols respectively). The third column: GO terms that the genes are annotated with. We only take into consideration GO terms in which this set of 10 genes is statistically significantly enriched. We only list GO terms that correspond to biological functions that the three drug mechanisms of interest rely on. BP denotes “biological process,” while MF denotes “molecular function” of GO. The fourth column: CVDs that the genes are associated with.

### The Core of Cardiovascular Diseasome

We apply the 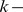
core decomposition algorithm to the human PPI network [24], [25]. The PPI network is iteratively pruned in search of its subnetwork in which all nodes are of degree at least 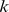. The steps of the algorithm are:

  1. All nodes of degree 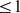, along with their edges, are removed from the network;
  2. In the resulting network, all nodes of degree 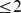, along with their edges are removed from the network;
  3. The process is repeated until only nodes of degree at least 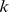 remain in the resulting pruned network. The largest value of 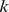 for which the resulting network is not empty is called 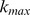, and the corresponding subnetwork is called 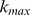 *-core*, or the *core* of the network.

The Core Diseasome is obtained purely computationally by computing the 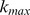-core decomposition of the human PPI network, along with the 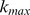-core decomposition of its subnetwork of only disease genes, described in [22]. Therefore, to investigate the importance of the 10 above described CVD related genes, we find the core of the human PPI network and check if these 10 genes are in it. Also we find the core of the PPI subnetwork consisting only of CVD related genes, and we check if this set of 10 genes appears in it (step 5. in Fig. 1). Since the core of the PPI network is known to contain driver genes and drug targets [22], we examine if any of the 10 genes are among the 15 known driver genes, or are drug targets [23], [38]–[40] (step 5. in Fig. 1). We obtain statistically significant findings (detailed in the Results and Discussion section), which allow us to postulate that these 10 genes are the *Key CVD Genes*. We further successfully validate this by checking the statistical significance of the overlap between Key CVD Genes and the Core Diseasome [22] (step 6. in Fig. 1).

### Predicting New CVD Genes

We use the above described method (steps 1–4 in Fig. 1) to predict novel CVD genes. We consider the 17 genes not currently known to be involved in CVDs, that are in clusters statistically significantly enriched in CVD genes, regardless of the value of the initial parameter 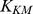. These genes are listed in Table 2.

**Table 2:** Predicted CVD genes.

Entrez ID	Gene name	GO term	Reference PubMed ID
1387	CREBBP	Receptor binding (MF), Signal transduction (BP).	14724353
4193	MDM2	Enzyme binding (MF).	18375498, 22821713
3065	HDAC1	Enzyme binding (MF).	22226905
4088	SMAD3	Enzyme binding (MF), Receptor binding (MF), Enzymelinked receptor protein signaling pathway (BP).	22167769, 22633655
4087	SMAD2	Enzyme binding (MF), Receptor binding (MF), Signaltransduction (BP), Intracellular signaling cascade (BP),Enzyme linked receptor protein signaling pathway (BP).	20829218, 22049534
3725	JUN, c-JUN	Signal transduction (BP), Response to drug (BP), Enzymelinked receptor protein signaling pathway (BP).	22664133
672	BRCA1	Enzyme binding (MF), Receptor binding (MF), Signaltransduction (BP), Intracellular signaling cascade (BP).	22186889
4609	MYC		22402364
6714	SRC	Signal transduction (BP), Intracellular signaling cascade (BP),Enzyme linked receptor protein signaling pathway (BP).	22287273
2033	EP300	Receptor binding (MF), Signal transduction (BP),Response to drug (BP).	20375365
7157	TP53	Enzyme binding (MF), Signal transduction (BP), Intracellularsignaling cascade (BP), Response to drug (BP).	23074332, 22189267
2885	GRB2	Receptor binding (MF), Signal transduction (BP), Intracellularsignaling cascade (BP), Enzyme linked receptor proteinsignaling pathway (BP).	12639989
8517	IKBKG	Signal transduction (BP), Intracellular signaling cascade (BP).	–
3320	HSP90AA1, HSP90AA2	Signal transduction (BP).	–
5295	PIK3R1	Enzyme binding (MF), Receptor binding (MF), Signaltransduction (BP), Intracellular signaling cascade (BP),Enzyme linked receptor protein signaling pathway (BP).	–
7543	YWHAZ	Signal transduction (BP), Response to drug (BP).	–
10971	YWHAQ	Signal transduction (BP), Intracellular signaling cascade (BP).	–

The first two columns: predicted CVD genes (Entrez Gene IDs and Official Gene Symbols respectively). The third column: GO terms that the genes are annotated with. We only take into consideration GO terms in which this set of 17 genes is statistically significantly enriched. We only list GO terms that correspond to biological functions that the three drug mechanisms of interest rely on. BP denotes “biological process,” while MF denotes “molecular function” of GO. The fourth column: if we validate that the predicted gene is associated with a CVD, we give the PubMed ID of the corresponding reference; “–”means that we found no literature validation.

**Figure 3:**
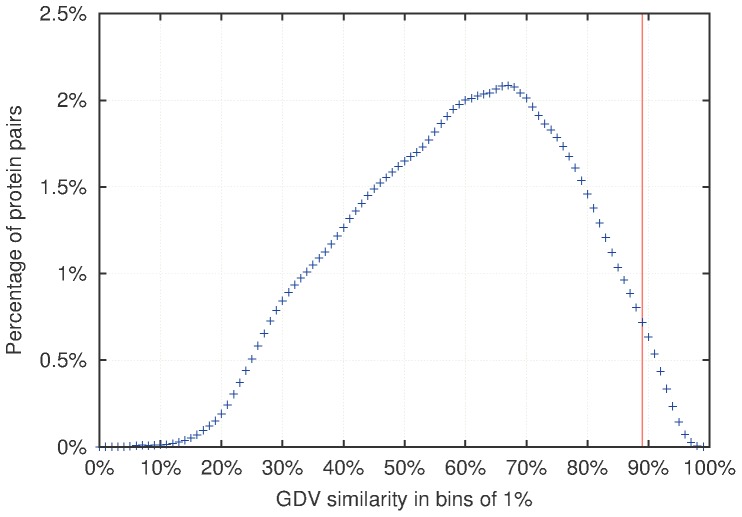
The distribution of GDV similarity of protein pairs in the human PPI network. Horizontal axis represents GDV-similarities of node pairs in the network in bins of 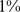. Vertical axis represents percentages of protein pairs that have a particular GDV-similarity.

Note that these 17 genes may have various GDV similarity to CVD genes, since all genes had to be assigned to clusters. Hence we seek only genes that are statistically significantly similar in topology to CVD genes. To do that, we compute the distribution of GDV similarities of all pairs of proteins in the human PPI network (Fig. 3). The top 1% of the most GDV-similar nodes have GDV similarity of at least 89% (corresponding to *p*-value of 0.01). Hence, amongst the 17 non-CVD genes, we look for those that are at least 89% GDV-similar to a CVD gene (step 7 in Fig. 1).

## Results and Discussion

**Figure 4:**
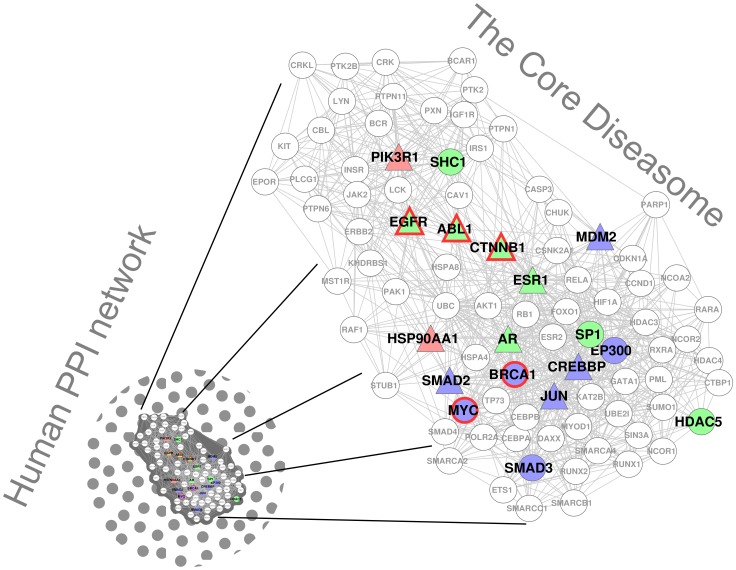
Summary of the results. The Core Diseasome of [22] is overlaid with the results of this study. Green nodes are the Key CVD Genes (from Table 1), which are in the Core Diseasome. Blue nodes are predicted CVD genes (from Table 2) that we validated in the literature and that are in the Core Diseasome. Red nodes are non-validated CVD gene predictions (from Table 2) that are in the Core Diseasome. Triangular nodes are drug targets. Driver genes are bordered in red.

Here, first we reason about the importance of the 10 CVD genes (listed in Table 1) identified by our methodology. Then, we validate our predicted CVD genes (listed in Table 2). Next we explain the therapeutic potential of the genes identified by our methodology. Finally, we provide a comparison with other approaches. The results are summarized in Fig.4.

### The Key Cardiovascular Disease Genes

We examine the importance of the 10 Key CVD genes as described in the section The Core of Cardiovascular Diseasome. We ask if they are in the 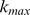-core of the PPI network and the 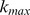-core of the PPI subnetwork of CVD genes only (steps 5–6 in Fig. 1), and if they are enriched in drug targets and driver genes.

We compute the 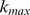-core decomposition of the PPI network: it consists of 372 proteins (recall that the entire PPI network has 7551 proteins). There are 44 genes in the intersection between these 372 proteins and the entire set of 423 CVD proteins in the PPI network. Interestingly, all 10 Key CVD genes, are among these 44 CVD -related genes that are in the core of the human PPI network. We calculate *p*-value for this to occur using the hypergeometric cumulative distribution with respect to entire human PPI network and with respect to 423 CVD-related genes. We find that both *p*-values are statistically significant, the first being 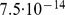 and the second being 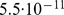. Furthermore, the connected subnetwork of the PPI network that consists only of CVD-related genes has 362 proteins, and its core consists of 43 genes. Again, all 10 Key CVD genes are in this core (*p*-value 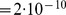 with respect to the 362 CVD proteins).

Also, three of the Key CVD genes: ABL1, CTNNB1, and EGFR, are among the 15 known driver genes. The two *p*-values, computed as described above are 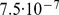 (with respect to entire PPI network), and 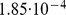
(with respect to 423 CVD genes).

We find that six out of the 10 genes are among the 1245 known drug targets that are present in the human PPI network. Table 3 lists Key CVD genes that are known drug targets and number of drugs from Drugbank that target the corresponding gene. Since 199 out of 423 CVD genes in PPI network are known drug targets, the *p*-value of getting 6 to occur amongst 10 Key CVD genes is not statistically significant. However, with respect to entire PPI network, this finding is statistically significant (*p*-value 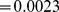).

**Table 3:** The Key Cardiovascular Disease Genes that are known drug targets.

Entrez ID	Gene name	Number of Drugs
367	AR	40
2099	ESR1	61
25	ABL1	11
1499	CTNNB1	1
2534	FYN	2
1956	EGFR	10

The first column: Entrez Gene ID. The second column: Official Gene Symbol. The third column: the number of drugs from Drugbank that target the corresponding gene.

Hence, we demonstrated the importance of 10 Key CVD genes.

As described in the Introduction, the Core Diseasome has been postulated to be the subnetwork of the PPI network that is the key to disease onset and progression and hence should be the primary object of therapeutic intervention [22]. Therefore we further validate the importance of our Key CVD Genes, by checking if they are a part of the Core Diseasome (step 6 in Fig. 1). We find that the following 8 out of the 10 Key CVD genes are in the Core Diseasome: SHC1, EGFR, ABL1, CTNNB1, ESR1, AR, SP1, HDAC5 (Fig.4). We check the probability for this or higher enrichment to occur purely by chance. This overlap is statistically significant with *p*-values of 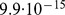 and 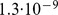 respectively (*p*-values computed as described in the beginning of this section). Note that GDV similarity measure is not necessary for the formation of the Core Diseasome, while the 10 Key CVD genes are obtained solely by using GDV similarity. Hence, validating the importance of Key CVD genes by checking their overlap with the Core Diseasome is not computationally biased.

### Validation of CVD Gene Predictions

We predict new 17 CVD genes, listed in Table 2, as the result of the same methodology that we used to identify the Key CVD genes (as described in the section Predicting New CVD Genes). We confirm that all of the 17 predicted genes are statistically significantly similar to some of the CVD genes.

To validate our predictions, we perform literature curation for possible CVDs that these 17 genes may be involved in. In the next section, we also examine therapeutic potential of these predictions.

We do the literature validations by text mining using CiteXplore (http://www.ebi.ac.uk/citexplore/): for the 17 predicted genes, we search PubMed abstracts with CiteXplore using their official gene symbols. In Table 2, we list the results of this literature mining and we discuss these findings below.

CREBBP gene is mentioned in connection with pathophysiological changes in cerebral vessels predisposing to stroke [41]. Gerzanich et al. [41] study three models of human conditions associated with stroke: chronic angiotensin II-hypertension, chronic nicotine administration and oxidative endothelial injury. All three models show significant up-regulation of expression of proliferative cell nuclear antigen (PCNA) in arterioles in situ, which is associated with increased activation of the nuclear transcription factor, phospho-cAMP response element binding protein (phospho-CREB).

It is shown that dilated cardiomyopathy tissues contain elevated levels of p53 and its regulators MDM2 and HAUSP (*p*-value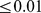) compared to non-failing hearts [42]. Also, regulation of MDM2 is critical in cardiac endocardial cushion morphogenesis during heart development [43]. Chen et al. [44] show that down-regulation of HDAC1 gene and the modifications on histone 3 lysine 4 (H3K4) and H3K9 significantly affect microRNA-29b expression in the context of signaling regulation of microRNA-29b, which is connected to novel mechanisms for cardiovascular diseases.

Aneurysms-osteoarthritis syndrome (AOS) is a newly discovered autosomal dominant syndromic form of thoracic aortic aneurysms and dissections, that is characterised by the presence of arterial aneurysms and tortuosity, mild craniofacial, skeletal and cutaneous anomalies, and early-onset osteoarthritis. AOS is caused by mutations in the SMAD3 gene [45]. It is known that aggressive cardiovascular phenotype of aneurysms-osteoarthritis syndrome is caused by pathogenic SMAD3 variants [46]. Also, SMAD2 dysregulation is associated with thoracic aortic aneurysms [47]. Inhibition of SMAD2 phosphorylation preserves cardiac function during pressure overload [48].

JUN gene is linked to different types of mitral valvular disease (MVD), including mitral regurgitation (MR) and mitral stenosis (MS) [49]. It is shown that c-Jun mRNA are significantly upregulated in patients with MS compared with those with MR (with *p*-value 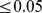) and that phosphorylated c-Jun N-terminal kinase in the MR group of patients is significantly greater than that in the MS group (with *p*-value 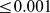).

It is demonstrated that proper expression of MYC in cardiac fibroblasts and myocytes is essential to cardiac angiogenesis, therefore MYC is required for proper coronary vascular formation [50]. It is shown that SRC protein regulates focal adhesion protein function, which influences contractility of vascular smooth muscle [51]. This also points to novel therapeutic approaches to CVDs, in terms of targeting SRC protein [51]. BRCA1 is an essential regulator of heart fuction [52]. BRCA1 and MYC are also driver genes [23](see Fig. 4).

Inhibition of EP300 can neutralize deficiency of KLF15 which is shown to be a molecular link between heart failure and aortic aneurysm formation [53].

It is known that TP53 is involved in cardiovascular functioning [54]. TP53 is also mentioned as one of the candidate genes associated with proatherogenic and inflammatory processes in chronic kidney disease (CKD) [55]. Zawada et al. aimed to point to new therapeutic strategies in CKD-associated atherosclerotic disease [55].

It is shown that GRB2 plays a role in the signaling pathway for cardiac hypertrophy and fibrosis [55].

For genes IKBKG, HSP90AA1, HSP90AA2, PIK3R1, YWHAZ, and YWHAQ, we found no evidence in the literature for their connection to cardiovascular diseases. However, due to the high literature validation score of our CVD gene predictions (over 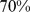 of our predictions are successfully validated in the literature), we predict that these genes are also involved in the processes related to cardiovascular diseases (step 8 in Fig. 1). Two of these genes (PIK3R1 and HSP90AA1) are part of the Core Diseasome, as shown in Fig. 4. PIK3R1 is associated with cancer and over-nutrition, while HSP90AA1 is associated with Alzheimer’s disease, cancer, eating disorder, herpes, and Fanconi’s anemia.

### Therapeutic Properties of Key and Predicted CVD Genes

The most common mechanisms by which drugs work are: (1) antibiotics, which disrupt bacterial cells causing them to die, or interfere with their essential reproduction machinery; (2) replacement drugs, which work by replacing substances missing from the body; (3) enzyme-acting drugs, which modify the enzymatic activity; (4) receptor-acting drugs, that either deliberately trigger cell surface receptors to activate the signaling machinery, or bind to those receptors to prevent ligands from performing their intended function; and (5) inter-cellular transport altering drugs, which modify the flow of molecules to and from a cell, thus changing their chemical composition and hijacking communication channels. Currently, therapeutic treatment of CVDs is achieved through drug mechanism types (3), (4) and (5) [57]–[59], while (1) is argued to have non-beneficial, or even harmful effects in treatment of CVDs [60]. This means that to be a CVD drug target, a protein would need to have a biological function that would facilitate the workings of the three above-mentioned drug mechanism types, (3), (4) and (5).

We use DAVID online tool (http://david.abcc.ncifcrf.gov/) to calculate Gene Ontology (GO) terms enrichments for the set of 17 predicted CVD proteins and the set of 10 Key CVD proteins. We upload each gene set separately to DAVID and use the entire set of human genes as a background set. We consider GO terms that correspond to enrichments that have *p*-values 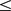 0.05 after the *Benjamini-Hochberg false discovery rate* (FDR) correction is applied. We find that the 10 Key CVD genes are statistically significantly enriched in the following GO terms which correspond to biological functions that the three drug mechanisms discussed above rely on: intracellular signaling cascade, intracellular receptor-mediated signaling pathway, signal transducer activity, and enzyme binding. We list these GO terms with their corresponding genes in Table 1. We find that the 17 predicted genes are statistically significantly enriched with the following GO terms which correspond to biological functions that the three drug mechanisms discussed above rely on: intracellular signaling cascade, signal transduction, enzyme linked receptor protein signaling pathway, response to drug, enzyme binding, and receptor binding. We list these GO terms with their corresponding genes in Table 2. We also check 199 known drug targets among CVD genes and find that they are statistically significantly enriched, with *p*-values 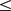 0.05, in biological functions that we list in Tables 1 and 2. This indicates that our methodology identifies important drug targets.

### Comparison with Other Approaches

Our methodology is based solely on network topology. In particular, we rely on GDV similarity between proteins in the PPI network. We compare it with baseline network topology based approaches to justify the use of GVD similarity for analyzing this particular dataset.

We examine clustering of proteins in the PPI network based only on the degrees (i.e. connectivity) of the nodes in the network. This method fails to identify any clusters statistically significantly enriched in CVD genes. Since guilt-by-association approach, based on protein interactors (neighbours) has become a relatively standard approach, we try to use it to identify “key” CVD genes. Hence, we look for statistically significant enrichment in CVD genes among the neighbours of each CVD gene in the network. There are 134 CVD genes that interact with sets of genes statistically significantly enriched in CVD genes. Therefore one may expect that these 134 CVD genes may be “key” for disease onset and therapy. Unfortunately this is not a case: this set of 134 genes is not statistically significantly enriched in the driver genes. Furthermore, it has no statistically significant overlap with the Core Diseasome and 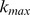-core of the PPI network. Hence, guilt-by-association can not be used to define Key CVD genes.

To verify that our methodology did not produce statistically significantly enriched clusters purely by chance, we randomized the topology of the PPI network respecting the degree distribution and performed the above described analysis on randomized networks (step 3 in Fig. 1). We repeated the randomization 30 times both for KM and HIE clustering. This did not yield any clusters statistically significantly enriched in CVD genes, which shows that specific topology around genes in the PPI network is a major contributor to identifying Key CVD genes and making predictions.

Note that analysis of all CVD genes and prediction of new ones has not previously been done using solely network topology. That is, our study is the first to use only topology to examine importance of CVD genes and predict new ones.

### Conclusion

This paper addresses an important, but difficult problem, and presents an approach that combines multiple methods in a novel way. We extract the Key CVD Genes that are enriched in drug targets and driver genes and that have a large overlap with the Core Diseasome.

We use our method to predict new CVD genes and validate a substantial portion of our predictions in the literature. Hence, it is likely that the remaining genes for which we did not find validation in the literature could be new genes involved in CVDs. Moreover, we find that the function of known CVD drug targets coincides with the function of many of our predicted CVD genes. This indicates that our method produces predictions that may be therapeutically exploited. Given the importance of CVDs to human health, even a small step in this direction may have substantial healthcare benefits. Biological validation and medical exploitation of our predictions, as well as characterization of key mechanisms responsible for disease formation and progression, are a subject of future research.
